# Interplay of Rice Stripe Virus and Rice Black Streaked Dwarf Virus during Their Acquisition and Accumulation in Insect Vector

**DOI:** 10.3390/v13061121

**Published:** 2021-06-10

**Authors:** Marcia Beatriz Moya Fernández, Wenwen Liu, Lu Zhang, Jamal-U-Ddin Hajano, Xifeng Wang

**Affiliations:** 1 State Key Laboratory for Biology of Plant Diseases and Insect Pests, Institute of Plant Protection, Chinese Academy of Agricultural Sciences, Beijing 100193, China; marciabeatriz806@gmail.com (M.B.M.F.); zhanglu13240106820@163.com (L.Z.); wangxifeng@caas.cn (X.W.); 2 Department of Plant Pathology, Faculty of Crop Protection, Sindh Agriculture University, Tandojam 70060, Pakistan; hajanojamal@gmail.com

**Keywords:** rice stripe virus (RSV), rice black-streaked dwarf virus (RBSDV), acquisition, accumulation, virus interaction

## Abstract

Plant viruses transmitted by hemipteran vectors commonly cause losses to crop production. Rice stripe virus (RSV) and rice black streaked dwarf virus (RBSDV) are transmitted to rice plants by the same vector, the small brown planthopper (SBPH), *Laodelphax striatellus* Fallén, in a persistent propagative manner. However, rarely do the respective diseases they cause occur simultaneously in a field. Here, we determined the acquisition efficiency of RSV and RBSDV when acquired in succession or simultaneously by SBPH. When RBSDV was acquired first, RSV acquisition efficiency was significantly lower than when only acquiring RSV. However, RBSDV acquisition efficiency from insects that acquired RSV first was not significantly different between the insects only acquiring RBSDV. Immunofluorescence assays showed that the acquisition of RBSDV first might inhibit RSV entry into midgut epithelial cells, but RSV did not affect RBSDV entry. SBPHs were more likely to acquire RBSDV when they were feeding on plants coinfected with the two viruses. When RBSDV was acquired before RSV, RBSDV titer was significantly higher and RSV titer first declined, then increased compared to when only acquiring RBSDV or RSV. Only 5% of the SBPHs acquired both viruses when feeding on plants coinfected with RSV and RBSDV. These results provide a better understanding of the interaction between two persistent viruses when present in the same vector insect and explain why RSV and RBSDV occur in intermittent epidemics.

## 1. Introduction

Rice stripe virus (RSV, genus *Tenuivirus*, family *Phenuiviridae*) and rice black streaked dwarf virus (RBSDV, genus *Fijivirus*, family *Reoviridae*) cause serious yield losses of rice in East Asian countries [1,2,3,4,5,6,7]. RSV usually causes chlorotic or necrotic stripes on newly developed leaves, then premature wilting and stunting, sometimes resulting in total crop loss [8,9,10]. RBSDV causes stunted stems and dwarfism in seedlings [11,12], especially severe growth retardation and excessive tillering, and typical rice grains do not form [13]. Although RSV and RBSDV are transmitted to host plants by the same insect vector, the small brown planthopper (SBPH, *Laodelphax striatellus* Fallén; order Hemiptera, family *Delphacidae*), diseases caused by these two viruses rarely occur simultaneously in the field [14]. A high viruliferous rate and population density of the vector insects are the main factors causing epidemics.

The SBPH is an important insect vector of several crop viruses due to its piercing and sucking mouthparts [3,15]. It does not migrate long distances and can complete its life cycle in a variety of Gramineae plants. The virus can then accumulate in an area once infection has been established. SBPHs can commonly be transported to other regions by wind or in infected seedlings. The favored stage for it to invade crops and transmit viruses is in the rice seedling stage. During unfavorable periods, SBPHs move to other cereals or weeds to survive and maintain their population [3,4]. During the winter, SBPH nymphs can survive on crops such as wheat, barley, and some grass weeds. Some insects can settle in wheat plants infected with RSV or RBSDV in the spring, then acquire the viruses to cause virus epidemics in rice or maize [16,17,18].

RSV and RBSDV are transmitted in a persistent and propagative manner in the SBPH [8,19,20,21,22,23]. Persistent viruses exist in the insect vector until its death, and some persistent viruses can replicate in the insect’s organs [19]. The virus firstly enters the insect gut and infects the epithelial cells where the virions replicate. Then, the virions move from the gut to other tissues, including the salivary glands from where they are transmitted to another plant to complete the horizontal transmission. Some persistent viruses such as RSV can be transmitted from female hoppers to their progeny via eggs (vertical transmission), but RBSDV is not transmitted to the next generation [24]. Insects have defense systems to avoid virus infection, replication, or release. Therefore, a virus that requires a specific insect vector for successful transmission needs to fend off attack by the insect’s immune system and overcome the transmission barriers in the midgut or salivary gland [19,25,26,27]. Effective transmission by the insect vector thus depends on factors in either the virus itself or the vector.

Thus far, the SBPH has rarely been found to carry both RSV and RBSDV in the field, but in laboratory experiments the insect can simultaneously carry both viruses [16]. However, little is known about whether RSV and RBSDV in same insect are favored or if the presence of one limits the acquisition efficiency of the other. In the present work, we studied the interplay of RSV and RBSDV when present in the same SBPH. We found that coinfection with RBSDV limited RSV acquisition by inhibiting RSV entry into the midgut epithelial cells of SBPHs and that only 5% of the insects acquired both RSV and RBSDV from coinfected plants. These results explain why diseases caused by RSV and RBSDV rarely occur simultaneously in the field. The results will also aid the development of more effective control measures and improve our understanding of the behavior of two persistent viruses that interact in the same vector insect.

## 2. Materials and Methods

### 2.1. Insect, Plant Virus, Virus Acquisition 

Second instar nymphs were used to assess acquisition of the viruses using three treatments with 100 nymphs per treatment. In the first treatment group (RBSDV-RSV), SBPHs were allowed to feed for 3 days on a plant infected with RBSDV, then for 3 days on another plant infected with RSV; the insects were then placed on virus-free rice seedlings for 7 days and checked for virus acquisition. The control insects fed only on the same RSV infected plant for 3 days. For the second treatment group (RSV-RBSDV), SBPHs fed for 3 days on a plant infected with RSV, then for 3 days on another plant infected with RBSDV. The SBPHs were then placed on virus-free rice plants for 7 days until the acquisition was checked and the control insects fed only on the same RBSDV infected plants for 3 days. For the third treatment group, insects were allowed to feed for 3 days on plants coinfected with RSV and RBSDV, then the insects were placed on healthy rice plants for 7 days to check for virus acquisition. The seedlings were grown in a growth incubator (27 °C, 16 h light/8 h dark). The experiment was done three times.

### 2.2. Total RNA Extraction and RT-PCR

Total RNA was extracted from individual insects and rice plants using TRIzol reagent and the manufacturer’s protocol, then resuspended in 20 µL of DEPC water and quantified with a NanoDrop 2000 spectrophotometer (Thermo Scientific, Waltham, MA, USA) as the ratio of OD260/OD280. Moloney Murine Leukemia Virus Reverse Transcriptase (M-MLV RT) from Promega, Madison, WI, USA was used to construct cDNA. For the PCR, 2 × M5 Taq H1F1 PCR Mix and specific primers were synthesized from BGI Tech (Bgi Tech Solutions Co., Ltd., Beijing, China). For RBSDV, forward primer One step RB F: 5′ AACAACCGACCAACAATCAC 3′ and reverse primer One step RB R: 5′ GAGCAGGAACTTCACGACAG 3′ were used, and for RSV, forward primer RSV-CP-F: 5′ ATGGGTACCAACAAGCCAGC 3′ and reverse primer RSV-CP-R: 5′ CTAGTCATCTGCACCTTCTGC 3′ were used.

### 2.3. Immunofluorescence Microscopy

Insects for each treatment were collected and placed on ice for 30 min, then dissected in a PBS solution to excise the guts. The guts were fixed in 4% (vol/vol) paraformaldehyde for 2 h, washed 3 times with PBS, and then incubated in osmotic buffer (2% (vol/vol) TritonX-100 in PBS) for 30 min at room temperature. After another wash with PBS, the guts were incubated with anti-RSV monoclonal antibody labeled with DyLight 549 (red), anti-RBSDV antibody labeled with Alexa Fluor 488 (green), and with phalloidin labeled with Alexa Fluor 633 in PBS containing 3% (wt/vol) BSA at 37 °C room temperature for 2 h. Samples were then washed three times in PBS, and stained with glycerol at room temperature. Fluorescence was observed with a Zeiss LSM 780 3-Channel Confocal Microscope [28].

### 2.4. RT-qPCR to Quantify Relative mRNA Transcript Levels

After checking the SBPHs which were infected with both viruses from the RBSDV-RSV groups and RSV-RBSDV groups by RT-PCR, we quantified the virus titer of RSV and RBSDV in the whole body of each insect by analyzing the expression of viral capsid genes (RSV pc3 and RBSDV p10) in the insect using RT-qPCR [29,30]. The relative RSV pc3 and RBSDV p10 RNA levels of the individual insects which fed on the virus source at 7 and 14 days after acquisition access to the second virus were calculated. For statistical analysis, we checked more than 10 insects for each group. The biological experiments were done at least three times independently. Total RNA was extracted from the insects using the standard TRIzol reagent protocol (Invitrogen). The purity and amount of extracted RNA were quantified using the NanoDrop 2000 spectrophotometer as the ratio of OD260/OD280. cDNA was synthesized from 2 μg of total RNA using a Fast Quant RT Kit (TIANGEN, Beijing, China). For an internal reference, the actin gene was used to normalize the quantity of total RNA purified from each sample. The Super Real PreMix Plus (SYBR Green) Kit (TIANGEN) was used for the PCR with a final volume of 20 μL containing 10 μL of PCR buffer, 0.5 μL of each primer (10 μM/μL), 1 μL of template cDNA, 7 μL of DEPC H_2_O, and 10 μL 2 × RealStar Green Fast Mixture ROX II. Real-time PCR was performed with SYBR Green using QuantStudio 6 Flex ThermoFisher (Applied Biosystems) and a thermocyling program of 95 °C for 2 min and 40 cycles of 95 °C for 15 s, and 60 °C for 30 s. Fluorescence was measured at the end of every 60 °C extension phase. Relative gene expression was calculated according to the Livak method (2^−ΔΔCt^).

## 3. Results

### 3.1. Acquisition Efficiency of RSV and RBSDV by SBPHs after Successively Feeding on Plants Infected with a Different Virus

**Figure 1:**
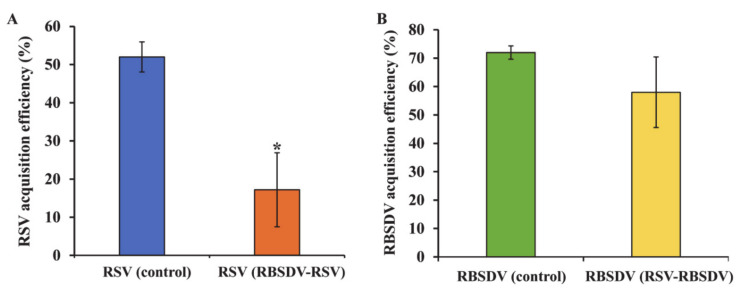
Virus acquisition efficiency after access of SBPH insect vector to RBSDV and RSV in different orders. Acquisition efficiency of (**A**) RSV after access to RBSDV first, then to RSV (RBSDV-RSV); (**B**) RBSDV after access to RSV first, then to RBSDV (RSV-RBSDV). The presence of viruses in individual insects was determined by RT-PCR using RSV- and RBSDV-specific primers. Asterisk (*) indicates a significant difference in efficiency of RSV acquisition between RSV-only control and RBSDV-RSV groups; *p* < 0.05 according to Tukey’s HSD test with 95% confidence.

When RBSDV was the first virus acquired by the insects, the acquisition efficiency of RSV (17%) was significantly lower than in the control insects that fed only on plants infected with RSV (52%) (Figure 1A), showing that RBSDV affects the acquisition efficiency of RSV by insects. However, when RSV was the first virus acquired, the acquisition efficiency of RBSDV did not significantly differ from the control insects that fed only on RBSDV-infected plants; thus, RSV did not affect later acquisition of RBSDV (Figure 1B).

### 3.2. Localization of RSV and RBSDV in Midgut Epithelial Cells from Insects Successively Feeding on Plants Infected with a Different Virus

**Figure 2:**
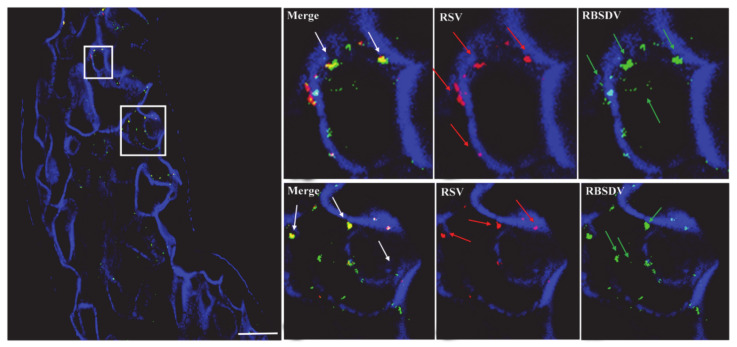
Localization of RBSDV and RSV in epithelial cells of midgut excised from third instar nymphs after RBSDV first–RSV second access. The image on the right are the amplification of the white squares. RSV was detected using Alexa Fluor 549 (red), RBSDV using Alexa Fluor 488 (green), and Alexa Fluor 633 phalloidin was used to label actin (blue). Scale bar, 20 μm.

**Figure 3:**
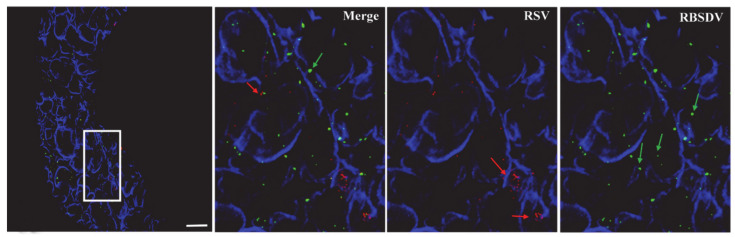
Localization of RBSDV and RSV in epithelial cells of midgut excised from nymphs after RSV first–RBSDV second access. The image on the right are the amplification of the white squares. RSV was detected using Alexa Fluor 549 (red), RBSDV using Alexa Fluor 488 (green), and Alexa Fluor 633 phalloidin was used to label actin (blue). Scale bar, 20 μm.

In the immunofluorescence assay to localize RSV and RBSDV in midgut epithelial cells of SBPH, numerous RBSDV virions were found on the exterior of the cell membrane and inside the epithelial cells of insects that fed first on plants infected with RBSDV and then with RSV, and RSV virions were only located on the exterior of the cell membrane, not inside epithelial cells (Figure 2). The result suggested that RSV virions could not enter the cells when cells were first infected with RBSDV. When RSV was the first virus acquired, RSV was located on the outside of the cell membrane and inside the epithelial cells, and some RBSDV virions were also on the cell membrane and inside the cell (Figure 3). Thus, cells that were infected with RSV were also invaded by RBSDV.

### 3.3. Viral Titer of RSV and RBSDV in Insects Successively Feeding on Plants Infected by Different Viruses

**Figure 4:**
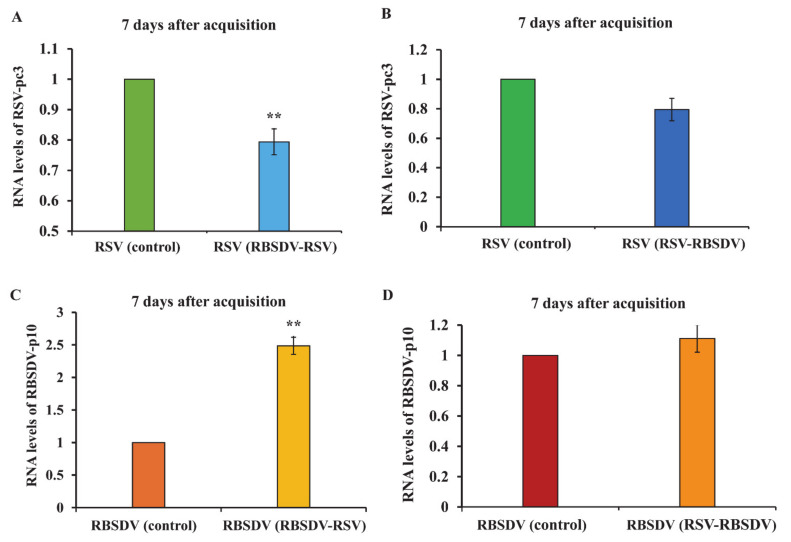
Relative RNA levels of RSV-pc3 and RBSDV-p10 at 7 days after acquisition access of RBSDV-RSV and RSV-RBSDV groups. RSV-pc3 relative RNA levels in RBSDV-RSV group (**A**) and RSV-RBSDV group (**B**); RBSDV-p10 relative RNA levels in RBSDV-RSV group (**C**) and RSV-RBSDV group (**D**). Three biological replicates were performed; each had three technical replicates. Asterisks (**) indicate a significant difference at *p* < 0.01 in Tukey’s HSD test with 95% confidence.

**Figure 5:**
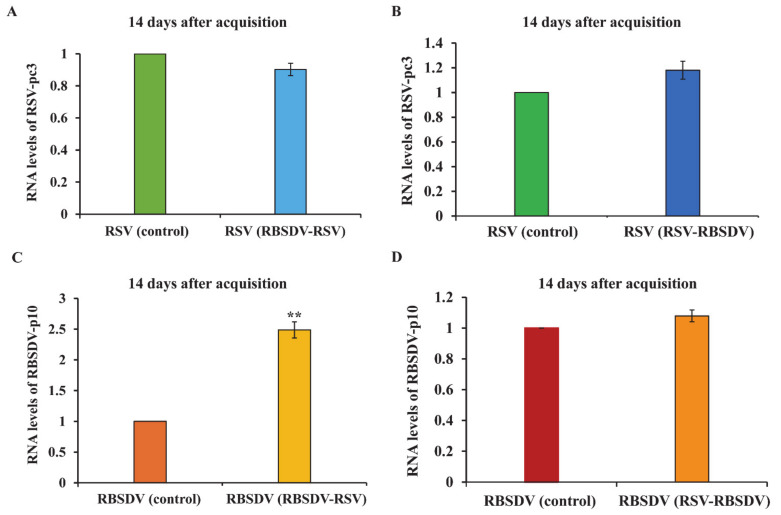
Relative RNA levels of RSV-pc3 and RBSDV-p10 at 14 days after acquisition access of RBSDV-RSV and RSV-RBSDV groups. RSV-pc3 relative RNA levels in RBSDV-RSV group (**A**) and RSV-RBSDV group (**B**); RBSDV-p10 relative RNA levels in RBSDV-RSV group (**C**) and RSV-RBSDV group (**D**). Three biological replicates were performed, each of which had three technical replicates. Asterisks (**) indicate a significant difference at *p* < 0.01 in Tukey’s HSD test with 95 % confidence.

After the two different acquisition orders (RBSDV-RSV and RSV-RBSDV), viral titer was quantified 7 and 14 days after the acquisition access to the second virus. The RT-qPCR showed that RSV-pc3 levels were significantly lower than in the RSV-only control insects after 7 days in the RBSDV-RSV group, but no significant difference in the RSV-RBSDV group was found (Figure 4A,B). However, expression of RBSDV-p10 was significantly higher than in the RBSDV-only control insects after 7 days in the RBSDV-RSV group, but also no significant difference in the RSV-RBSDV group was found (Figure 4C,D). After 14 days of acquisition access to the second virus, only RBSDV-p10 RNA levels were significantly higher than in the RBSDV-only control insects in the RBSDV-RSV group (Figure 5). This suggested that when RSV was acquired first by the vector insect, the levels of RBSDV-p10 and RSV-pc3 were not significantly different compared with the RSV- or RBSDV-only controls after 7 or 14 days. However, RBSDV-p10 RNA levels were significantly higher than in the RBSDV-only control insects after both 7 days and 14 days when RBSDV was acquired first. 

### 3.4. Acquisition and Accumulation of RSV and RBSDV in SBPHs That Fed on Plants Coinfected with Both Viruses 

**Figure 6:**
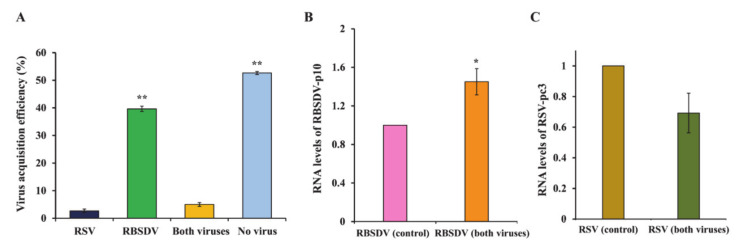
Acquisition and accumulation of RSV and RBSDV after 3-day acquisition during a period of access to a plant coinfected with the two viruses. (**A**) Acquisition efficiency of RSV and RBSDV. Relative expression levels of (**B**) RBSDV-p10 and (**C**) RSV-pc3. Three biological replicates were performed; each had three technical replicates. Asterisks indicate a significant difference at (**) *p* < 0.01 and (*) *p* < 0.05, according to Tukey HSD test with 95 % confidence. Asterisks indicate a significant difference at (**) *p* < 0.01 and (*) *p* < 0.05 in Tukey’s HSD test with 95% confidence.

The RT-PCR to detect the viruses in the insects showed that, after 3 days of acquisition access to coinfected plants, around 50% of the insects did not acquire virus, 39% acquired RBSDV, only 3% acquired RSV, and 5% acquired both (Figure 6A). Further, when RSV-pc3 and RBSDV-p10 RNA levels were detected by RT-PCR, RBSDV-p10 levels were significantly higher than in the RBSDV-only control insects (Figure 6B). Although the levels of RSV-pc3 were low, they did not differ significantly from the RSV-only control levels (Figure 6C).

## 4. Discussion

Acquisition by a vector insect is the first and critical step in the transmission of arboviruses [20]. Previous studies showed that SBPH nymphs can acquire viruses more effectively than the adults can [18,19,31]. Therefore, we used nymphs of the second instar in our work to acquire high efficiencies for RSV and RBSDV [1,6,14,32]. Although an acquisition period of 2 days is often used, we found it was insufficient for the insects to acquire both viruses. Further, RSV acquisition efficiency by SBPHs is positively correlated with the duration of the acquisition [33]. The acquisition efficiency of RSV was 36.7% after 2-day access to leaves freshly infected with virus [1]. In our study, the efficiency for RSV acquisition was higher (52%) after 3 days for the acquisition, which indicates that the longer the acquisition duration, the greater the acquisition efficiency of the insect vector. The efficiency of RBSDV acquisition by SBPHs after 3 days was also higher than after 2 days [34]. 

Our studies showed that after the insects had acquired RBSDV, the acquisition efficiency of RSV was significantly lower than in the RSV-only controls, but the acquisition of RSV first did not affect the acquisition efficiency of RBSDV. As the midgut is the most difficult and important barrier that the virus has to cross [19,25,26,35,36], we used immunofluorescence to examine the insect midgut epithelial cells and found that RSV had limited entry into the epithelial cells when RBSDV was acquired first. Sugar transporter 6 protein (LsST6) of SBPHs is highly expressed in the midgut and mediates the entry of RSV into the epithelial cells. LsST6 also interacts with the outer capsid protein of SRBSDV [22]. Therefore, RBSDV might bind LsST6 to inhibit the interaction of RSV and LsST6, so that RSV cannot enter the cells. However, we found that infection with RSV first did not limit RBSDV entry into gut epithelial cells, indicating that RBSDV might infect cells in ways that are not affected by RSV.

Arboviruses commonly assist or limit the transmission of each other when they coinfect the same insect vector [16,26]. When RSV infected the insect first, the titer of RSV and RBSDV was not affected. In a previous study that analyzed the siRNA level of both viruses, RSV and RBSDV did not affect the accumulation of the other virus in RSV-infected insects after they acquired RBSDV [14]. Thus, insects that acquire RSV first showed no significant effect on the accumulation of RBSDV, as we showed previously (Figure 5). However, it was interesting that the viral titer of RBSDV was significantly higher in the insects that were infected first with RBSDV, then with RSV. As for coinfection with other plant viruses, each usually inhibits the acquisition or transmission of other viruses in the same insect vector. For example, coinfection with a wheat rhabdovirus causes a significant reduction in the Mal de Río Cuarto virus (MRCV) titer in their planthopper vector (*Delphacodes kuscheli*), suggesting that the presence of the rhabdovirus impairs the acquisition or replication of MRCV in the same insect [26]. Tomato yellow leaf curl virus (TYLCV) and tomato spotted wilt virus (TSWV) are two persistent viruses transmitted by *Bemisia tabaci* Q and *Frankliniella occidentalis*. Although *Bemisia tabaci* Q cannot transmit TSWV, but it is able to acquire and retain TSWV, the acquisition, retention, and transmission of TYLCV by *Bemisia tabaci* Q were reduced when the insect vector contained TSWV. The transmission of TSWV by *Frankliniella occidentalis* was also reduced when the insect contained TYLCV. This demonstrated that persistently transmitted viruses can restrict the transmission of other viruses by affecting insect vector [16]. However, our results showed that the entry of RSV as the second virus might facilitate the replication of RBSDV. Although the mechanism by which RSV helps RBSDV replication is not clear, this phenomenon suggested that coinfection with the two plant viruses may promote the accumulation of one persistent virus in the insect vector. Thus, RBSDV restricts the entry of RSV because a large number of virions would be detrimental to insects.

After SBPHs fed on plants coinfected with RSV and RBSDV, the acquisition efficiency for RBSDV (39%) was significantly higher than for RSV (3%), indicating that the insects were more likely to acquire RBSDV when both viruses were present. The potential for acquiring both viruses at the same time was low, occurring in 5% of the insects, probably because the insects acquired RBSDV first, limiting RSV infection. These results could explain why it is rare for RBSDV and RSV to coinfect the same insect in the field. The competence for the acquisition of a virus is determined by interaction between the specific components of a virus and its insect vector [20]. There is a close relationship between the viral titer in the insect vector *Thrips tabaci* and the transmission of tomato spotted wilt virus (TSWV); viral titer is higher in transmitting insects than in non-transmitters [37]. The selective behavior of SBPHs after the coinfection in the present study favored the acquisition and accumulation of RBSDV but not RSV. The acquisition of viruses by their insect vectors is a process dependent on the insect responses and on the retention and circulation capacity of the virus in the vector’s body [38]. These effects could favor the capacity of RBSDV transmission by the insect vector. On the other hand, studies on the coinfection of plants with two potyviruses (watermelon mosaic virus (WMV) and zucchini yellow mosaic virus (ZYMV)) and on the behavior of aphids when feeding on different combinations of mixed infections in plants showed greater virus assimilation by aphids (*Aphis gossypii*) from coinfected plants than from plants with single infections [39]. ZYMV was found to be deficient in an auxiliary component protein that functions in the binding of the virion to the aphid’s mouth parts [40,41]. Therefore, studies are needed to determine why the SBPH acquires more RBSDV than RSV.

In conclusion, we found that RBSDV limited RSV acquisition in their insect vector. The SBPHs were most likely selective for RBSDV and had low capacity to acquire RBSDV and RSV from coinfected plants, explaining why it is rare to find SBPHs with both RSV and RBSDV in the field. The SBPH is the only natural vector of RSV and RBSDV and efficient transmission by the SBPH vector is required for epidemics caused by the two viruses. Therefore, a better understanding of how the two viruses reciprocally affect acquisition and accumulation by the same insect vector will help dissect the underlying causes of the intermittent epidemics of these viruses. 

## Data Availability

Not applicable.
